# Technostress and time spent online. A cross-cultural comparison for teachers and students

**DOI:** 10.3389/fpsyg.2024.1377200

**Published:** 2024-07-10

**Authors:** Ana-Maria Cazan, Laura Teodora David, Camelia Truța, Cătălin Ioan Maican, Ramona Henter, Laura Elena Năstasă, Niko Nummela, Olli Vesterinen, Arne Morten Rosnes, Tobias Tungland, Eirin Gudevold, Mari Digernes, Dagmar Unz, Stefanie Witter, Mariela Pavalache-Ilie

**Affiliations:** ^1^Faculty of Psychology and Education Sciences, Transilvania University of Brasov, Brasov, Romania; ^2^Institute of Philosophy and Psychology “Constantin Rădulescu-Motru”, Romanian Academy, Bucharest, Romania; ^3^Faculty of Economic Sciences and Business Administration, Transilvania University of Brasov, Brasov, Romania; ^4^Doctoral School of Economic Informatics, Bucharest University of Economic Studies, Bucharest, Romania; ^5^RDI Department, Diaconia University of Applied Sciences, Helsinki, Finland; ^6^School of Vocational Teacher Education, Haaga-Helia University of Applied Sciences, Helsinki, Finland; ^7^Section for Teaching Support and Digital Development, VID Specialized University, Oslo, Norway; ^8^Faculty of Applied Social Sciences, THWS Technical University of Applied Sciences Würzburg-Schweinfurt, Würzburg, Germany

**Keywords:** technostress, time spent online, optimal use of technology, technology self-efficacy, social support

## Abstract

**Introduction:**

Research shows that ICT is beneficial for academics and students, aiding in overcoming distance barriers, streamlining administration, and improving teaching and learning processes. However, the negative impact of technology, particularly technostress, are garnering attention. In the context of the concerns about technostress among higher education institutions (HEI), the aim of the study is to analyze the technostress creators and inhibitors for university teachers and students in different European countries. The topical concept of digital well-being is seen as “a subjective individual experience of optimal balance between the benefits and drawbacks obtained from mobile connectivity, focusing on the personal perception of what amount of time spent using technology is optimal so that well-being is preserved”.

**Method:**

To explore specific aspects related to use of technology, two – parallel online surveys for academics (*N* = 446) and students (*N* = 660) from four European countries (Romania, Germany, Norway, and Finland) were conducted between November 2022 – January 2023. The surveys included the Technostress scale and the Technostress Inhibitors Scale, the Technology self-efficacy Scale, and a questionnaire focusing on socio-demographic aspects, work experience, academic field, dimensions related to the actual use of technology and participants perception on the optimal use of technology for work, learning or personal tasks, in terms of the period of day/week and amount of time spent. We also inquired about the social support given and received when using technology and the formal and informal rules, expectations, policies, punishments, and rewards regarding the use of technology.

**Results:**

The findings suggest that the perceived optimal use of technology is significantly lower than the actual use for all the contexts. Overuse of technology was associated with technostress. Our results also showed that technology self-efficacy and social support from colleagues and teachers are negatively associated with technostress. Country differences regarding technostress and time spent online were also observed.

**Discussions:**

Despite the needed caution in interpreting the results because of the unbalanced sample size across countries, the results could be used to develop research and support interventions within European countries to promote digital well-being, a better work-life balance with further positive effects on academic satisfaction and work/learning productivity.

## Introduction

We are living in time of the fourth industrial revolution which means that our daily life and our professional activities are engulfed by digital tools and information and communication technologies (ICT) from the way we pay our groceries to the solutions that are installed in order to solve medical problems such as gene editing. Apps and artificial intelligence as prompters to our most routinely actions are a fact and the pace that changes are made in technologies is alert.

Digitalization defined as “the manifold sociotechnical phenomena and processes of adopting and using (…) [digital] technologies in broader individual, organizational, and societal contexts” (Legner et al., 2017, p. 301) has penetrated almost every aspect of human activities and brings challenges as well as development. Positive consequences such as improving people’s quality of life (Kryzhanovskij et al., 2021) and supporting economic growth and business development (Parviainen et al., 2017; Bloomberg, 2018) are obvious. Flash Eurobarometer published in April 2022 (Leclerc et al., 2022) looked at how much European citizens are using digital technologies in the workplace and also at their physical and mental health, exploring the adverse effects of technology. More than 27,000 participants offered answers and 52 % of respondents answered that the use of digital technologies in their workplace determined the speed or pace of their work, with 33% considering that these technologies increased their workload.

There is extensive data on digitalization and its impact on professional activities in general, but more knowledge is needed on the impact of technology use in HEI as universities are incubators of future work force.

Research shows that ICT is beneficial for academics and students, aiding in overcoming distance barriers, streamlining administration, and improving teaching and learning processes. However, the negative impacts of technology, particularly technostress, are rising attention. Technostress, defined as the inability to cope with new technologies, affects various groups including higher education employees and students (Tarafdar et al., 2015; Hauk et al., 2019) being also linked to decreased productivity and other negative outcomes (Truța et al., 2023). While there’s extensive research on technostress among employees, there are only several empirical studies focusing on its prevalence among academics and students after the pandemic. Understanding technostress in this context is crucial, as it may lead to reduced academic productivity and other negative consequences in HEIs (Upadhyaya and Vrinda, 2021). But not always the effect of technostress is negative. University employee’s performance and quality of work life while using digital technology is increased by higher organization flexibility and offered support such as training and peer assistance even if the staff is experiencing technostress (Saleem et al., 2021; Saleem and Malik, 2023).

Other studies focusing on technostress in academia (Truța et al., 2023) emphasize the associations with self-efficacy, personality traits, or the negative effects on well-being (Smith and Ulus, 2020) and motivation (Vallone et al., 2023), solely on employees samples. Most of these studies use self-report measures of technostress, assessing the subjective perception of individuals on the experienced impact of technology use. There is little evidence about technostress in relation with the actual use of technology in terms of time spent online. For example, Salanova et al. (2013) investigated two dimensions of technostress, namely technostrain and technoaddiction, on samples of intensive ICT users and non-intensive ICT users, this being one of the few studies in the literature which takes the intensity of ICT use at work into account.

In the context of the concerns about technostress among HEI’s, the aim of the study is to analyze university teachers’ and students’ technostress in different European countries, in the context of the growing trend of digitalization in academia. The identification and description of technostress and of the factors that develop or mitigate technostress could help policymakers to implement programs fostering academics’ and students’ well-being. The changes brought about by digitalization cannot be undone and should not be therefore we need to build a positive perception of it by focusing our attention on what influences this perception and on the strategies that can be used to make changes when necessary. The research aims to address the following research questions:

- Are there differences between what is considered optimal use and the effective use of technology, for students and academics?
- Is there a significant difference between the number of received demands and the solved and initiated demands and could this be considered a sign of overload?
- Are there associations between technostress and various aspects regarding the use of technology?

## Literature review

The focus of our research is higher education institutions (HEI) agents, namely academics and students who are no strangers from digitalization and ICTs. Many rapid changes in how HEI responded to technology immersion were noticed during and shortly after the pandemic period while activity in HEI was disturbed by restrictions. It has become clear that digitalization is a complex phenomenon with both positive and negative outcomes. On the one hand it encourages learning of new skills, use of IT equipment, use of apps and digital solutions that facilitate flexibility, track progress and make feedback more visible and available. On the other hand, there were difficulties in adapting and transforming face to face traditional learning experiences in online ones. Increased pressure on both teacher and students might affect their well-being and feelings of self-efficacy. Above all, access to technology and adequate technical support became a necessity that was not always met. The use of technology is a must as a tool both in teaching and in learning processes, but it is also needed in research activities that is a compulsory component of teachers’ activity in HEI (Moncayo Martínez et al., 2020; Kutsak et al., 2023). Apart from teaching and researching, academics are also involved in so called administrative tasks, meaning they have to contribute to secretarial demands linked to their teaching and researching activities. Students, on the other hand, receive academic demands that are posted online, are asked to review information and contents in different format and to do homework and school projects that require digital tools. It is clear that neither academics nor students can avoid computers or digital platform while working.

Technology may impact individual’s lives either by enhancing work engagement and productivity or by invading personal life. There is a fine line between the two and personal perception is what describes it best. The Person–Technology (P–T) fit model, derived from Person–Environment fit model, is used to survey the match between person and technology to better understand the effect of technology use on people (Ayyagari et al., 2011).

Technology can bring about both positive and negative outcomes (techno-eustress and techno-distress) depending on one’s attitude and perception of connectivity through digitalization. To facilitate the eustress and mitigate the techno-distress, emotional regulation strategies and IT resilience can be developed to help professors and students maintain their subjective well-being. The topical concept of digital well-being is seen as “a subjective individual experience of optimal balance between the benefits and drawbacks obtained from mobile connectivity” (Vanden Abeele, 2021, p. 938), focusing on the personal perception of what amount of time spent using technology is optimal so that well-being is preserved.

Hence, the time spent online was identified as the variable congregating the types of technostress creators and inhibitors that can be met in academia because one’s ability to regulate the time and type of activities carried on in the digitalized area of their work may impact their perception on technology use. We did not set a margin for what is optimal use of technology and what is not in terms of time allotted, or activities carried out, but we let the participants express their opinion on this.

The unavoidable situation introduced in society by COVID-19 restrictions worked both as a wake-up call for HEI stakeholders and as a trigger to generate change (Nikou and Aavakare, 2021; Fűzi et al., 2022), so much so that digital competences are mandatory requisites for both academics and graduates. The very nature of digital technology is introducing new format and upgrading continually in order to respond better to an overwhelming number of data that technology itself is creating. The high pace of technology development induces stress in users because one has to keep being informed with all these changes (Ragu-Nathan et al., 2008; Tarafdar et al., 2015).

Using technology as a job demands implies the need not only for training and organizational support (Ayyagari et al., 2011; Saleem et al., 2021) but also inner resource especially a sense that you are able to use technology in an effective manner and trust that you have capabilities elicited by professional tasks. These capabilities are underlined by the technology self-efficacy construct, mentioned by Chou and Chou (2021) as being negative correlated with technostress and by Woolston (2019) as an indicator of openness to digitalization. Higher level of technological self-efficacy prone people to use more apps and decrease the level of technostress (Truța et al., 2023).

Technostress means the inability to cope with new computer technologies in a healthy manner (Tarafdar et al., 2015). The larger concept includes five types of technostress creators and three type of technostress inhibitors (Tarafdar et al., 2015). Techno-overload refers to the increased number of tasks and errands that an employee has to respond to, the flow being fast and ongoing. Techno-invasion signifies the expectation to remain always available online, to check notification and keep activity logs that track all of the activity no matter if is work related or not. It may also include surveillance issues due to track location options, assaulting sometimes the privacy of one’s home or free time. Techno-complexity denotes the unceasing progression of technology and the pressure to keep up with the changes that cost energy and time from users. They might experience being overwhelmed and fear being left behind, so they tend to push themselves in order to ensure the required proficiencies. Techno-insecurity is generated by the advance in technology that might take over human tasks and leave people without work. Techno-uncertainty describes anxiety caused by the concern that something has changed in how technology tools work without one being aware of or because of being always on edge about policy change regarding ICT at the workplace or responsibility that might be revoked or added any moment. Techno-stress inhibitors refer to literacy facilitation, technical support provision, and involvement facilitation that are felt by a person after taking into consideration not only internal factors (such as their own skills) but also external factors and access to support provided either by employee or by other significant agents.

A large body of literature regarding how technostress is perceived by main actors of HEI showed that younger students tend to experience lower levels of technostress compared with their teachers, and age of teachers positively correlates with the level of technostress (Hauk et al., 2019) technostress having a negative impact on teacher’s life and work performance. Technostress negatively impacted teacher’s life and performance (Aktan and Toraman, 2022) especially if there is a lack of flexibility regarding professional requirements at organizational level (Saleem and Malik, 2023). Female students tend to feel higher technostress (Upadhyaya and Vrinda, 2021). Regarding educational background, the higher the education level, the lower the technostress level (Li and Wang, 2020; Upadhyaya and Vrinda, 2021). When students perceived high levels of technostress overload and low level of personal technical abilities, they also appreciated online learning environment as less satisfying (Conrad et al., 2022). Mushtaque et al. (2022) mentioned a negative association between technostress and online format of the instruction reported by students in medicine. The relations between technostress and instructional process is not always straight forward as students are not alone in the educational context. Saleem et al. (2024) showed that support from the teacher and from the university mitigate the negative impact of technostress on quality of online learning in students.

Discussing which facet of the technostress contributed more to the negative emotions, the results were not consistent (Asad et al., 2023). Techno-insecurity was found to have a great impact on psychological well-being (Asad et al., 2023), while Upadhyaya and Vrinda (2021) concluded that techno-invasion and techno-overload were the larger contributors to technostress in students. The results of a study (Qi, 2019) looking at the reason of using technology in an attempt to compare if the same unpleasant feeling was associated with technology if is used for school task of for leisure activities were not conclusive.

The deployment of ICT can lead to constant connectivity, where employees feel the need to be available and responsive beyond traditional working hours. This situation can result in work encroaching on personal time, leading to stress and burnout (Curcuruto et al., 2023). Therefore, analyzing the balance between what academics consider optimal use of technology and actual use could offer insights about the sources of technostress and the role of possible stressors, such self-efficacy and social and organizational support to mitigate the negative effects of technostress.

Our study bridges the academia’s and students’ perceptions on what could be defined as an optimal use of technology in order to benefit from it with what they feel is actually happening in their workplace or learning place at the moment so that further measures could be taken to reduce technostress. We are concerned not only with the employees’ perception, but also the students’ as there is an intrinsic connection between teachers and students and their success in their work depends on each other’s.

## Methods

### Participants

Data were collected online using the Lime Survey (open source). The two versions of the surveys – one for academics and one for students - were initially constructed in English and then translated and adapted into the national languages of each of the four countries included in the study. The links to the translated and adapted surveys were sent to institutional email addresses of academics and students from different universities in the participating countries. No rewards were involved for participating in the study, and for all participants confidentiality of responses was guaranteed. Data was collected between November 2022 – January 2023.

Academic sample: The participants were 446 academics, from four countries: Romania (45.1%), Finland (16.1%), Norway (33.6%), Germany (5.2%). 22.9% of the participants had a management position. The sample covered several academic fields: Arts and Humanities (11.5%), Engineering (22.9%), Medicine (16.1%), Natural and applied sciences (6.3%), and Social sciences (43.2%).

Student sample: The participants were 660 students, from four countries: Romania (61.4%), Finland (10.6%), Norway (18.8%), Germany (9.2%). Regarding their employment, 178 declared they were employed part-time (*N* = 98) and full-time (*N* = 80). Two hundred and forty-five students are undergraduates. The sample covered several academic fields: Arts and Humanities (11.3%), Engineering (14.9%), Medicine (13.8%), Natural and applied sciences (13.2%), and Social sciences (46.8%).

### Measures

For both the academics’ and the students’ samples, the survey consisted of several items measuring socio-demographics data, standardized scale for measuring technostress, technostress inhibitors, and other variables related to use of technology in various academic context.

- The socio-demographic questionnaire including questions related to: nationality, type of job (teaching, research, administrative), work experience, academic field, and university for the academics. For the student sample, the questionnaire included questions related to: nationality, type of student (undergraduate, graduate, PhD student), academic standing and academic field, faculty and university, and employment status.
- Technostress was measured using the 12-item Technostress scale (Tarafdar et al., 2015), measured on a 5-point Likert scale (strongly disagree to strongly agree), Cronbach’s Alfa for the entire scale being 0.89 for the academics’ sample and 0.87 for the student sample. It measures three technostress creators using 5-items scales: Techno-overload (5 items, Cronbach’s Alpha coefficient of 0.91/0.85), Techno-invasion (4 items, Cronbach’s Alpha coefficient of 0.81 for the academics and 0.78 for the student sample), Techno-complexity [3 items, Cronbach’s Alpha coefficient of 0.82 (academics)/0.83 (students)]. Examples of items: I am forced to change my work (learning) habits to adapt to new technologies; I spend less time with my family due to this technology; I often find it too complex for me to understand and use new technologies.
- Technostress inhibitors were measured using the Technostress Inhibitors Scale (Tarafdar et al., 2015) [13 items, measured on a 5-point Likert scale (strongly disagree to strongly agree, Cronbach’s Alpha = 0.90 both for academics and students)] using 5-items scales. The scale conceptualizes technostress as being manifested in the three dimensions: Literacy facilitation [5 items; Cronbach’s Alpha = 0.87 (academics)/0.85 (students), Our university provides end-user training before the introduction of new technology], Technical support provision [4 items; Cronbach’s Alpha = 0.91 (academics)/ /0.89 (students), The IT department in our university is well staffed by knowledgeable individuals] and Involvement facilitation [4 items, Cronbach’s Alpha = 0.78 (academics)/0.78 (students), We are encouraged to try out new technologies].
- Technology self-efficacy was measured using the five-item Technology self-efficacy Scale (Venkatesh et al., 2003; Gu et al., 2013) with Cronbach’s Alpha 0.89 for academics and 0.90 for students. Examples of items: Whether the use of online technology is difficult or easy, I am sure that I can understand it; I feel confident that I have the necessary skills to use online communication and collaboration applications. The five items were measured on a 5-point Likert scale (strongly disagree to strongly agree).

Both technostress scales (technostress creators and technostress inhibitors) and technology self-efficacy were previously used on HEI samples, showing excellent reliability (Truța et al., 2023).

- Dimensions related to the use of technology for work, learning or personal tasks, during weekdays or weekends were also added, the items asking the participants to estimate the amount of time they spent engaged in (compulsory and non-compulsory) job-related tasks (How many hours a day do you spend using technology for: job-related (compulsory and non-job compulsory): Teaching/Research/Administrative tasks, weekdays and weekend day and non-job related). The frequency of use (8 items) and perceptions about optimal use [4 items, How many hours a day do you regard as an optimal use for job related activities (Teaching/Research/Administrative tasks)?] were measured. For students, the items asked participants to estimate the amount of time they spend engaged in school-related (compulsory and non-compulsory) tasks. The frequency of use [3 items, How many hours a weekday/weekend day do you spend using technology for: school-related (compulsory and non-compulsory) activities?] and perceptions about optimal use (2 items, How many hours a day do you regard as an optimal use for school-related activities) were measured. These were presented to the users using either number boxes or dropdown lists with predefined values.
- Social support was measured with two items on a10-point Likert scale, ranging from never to always, How often do you get help and support from your colleagues? and How often do you get help and support from your nearest superior? inspired by (Pejtersen et al., 2010).
- Formal and informal rules, expectations, policies, punishments, and rewards regarding the use of technology (Piszczek, 2017) were measured with several items grouped in two dimensions: communications that occur outside regular working hours (5 items, How many work-related demands did you receive after hours in an average week?) and expectations about availability (8 items, Cronbach Alpha = 0.93 My university expects me to answer after-hours contacts immediately). For students, formal and informal rules, expectations, policies, punishments, and rewards regarding the use of technology were measured with 5 items focused on university-related demands (university-related demands: messages, emails, phone calls, etc.) received/ initiated etc. The items are measured on a 5-point Likert scale, ranging from 1 not at all true to 5 completely true.

### Data analysis

To analyze the data, nonparametric tests (Wilcoxon and Friedman tests for repeated measures and Spearman correlations) were used for the analyses involving the number of hours spend using technology, the number of received, initiated and solved demands, all these variables being non-normally distributed (positive asymmetry). Different nonparametric tests were used for academic and student sample because the number of compared contexts was also different (Teaching activities, Research activities, Administrative tasks, Non-Job activities for academics versus Academic tasks, Non-academic tasks for students). Friedman’s ANOVA compares the difference between more than two related measures, while Wilcoxon Test is used to compare two related measures (Kim, 2014).

The psychological dimensions, such as technostress, technology self-efficacy, and social support were normally distributed, with skewness values between −0.79 and 0.67 and kurtosis values between −0.45 and 1.03. Pearson correlations were computed for the associations between variables and One Way ANOVA for country differences.

In addressing common method bias in our study, several effective strategies were implemented to enhance the validity and reliability of the research findings. Psychometric separation was used to mitigate issues associated with using a single format or scale, our study employed diverse methods for collecting responses. This approach was addressed by using a range of response formats across different questions to capture a broad spectrum of data. Specifically, some questions required numerical inputs, while others used a Likert scale ranging from 1 to 5 (more details were provided in the Measures section), by extending beyond traditional scales and incorporating scales ranging from 0 to 10 and dropdown lists, further diversifying the response options. The online form for collecting data also included questions distributed across multiple pages within a larger study framework, which helps in reducing respondent fatigue and the potential for patterned responses. To address social desirability bias and encourage more honest responses, the study emphasized the anonymity and confidentiality of participant data. Key measures included: utilizing a form-building application that anonymizes responses, ensuring that no personally identifiable information, such as email addresses was collected. This approach likely helped in reducing any hesitation participants might feel about providing responses, thus enhancing the authenticity of the data collected. In addition, by gathering data from varied sources, the study aimed to compare and control for common method variance effectively. Specific measures included: administering a multi-language questionnaire across different institutions, which helps in capturing diverse perspectives and reduces the bias that might arise from a single linguistic or cultural background; separating data collection between different respondent groups (teachers/employees vs. students) and conducting these sessions at different times further minimized the risks of common rater effects and temporal biases. All these approaches were carefully included to adequately deal with common method bias, enhancing the study’s integrity by ensuring a more accurate representation of the relationships among the variables investigated.

## Results

### Are there differences between the effective use of technology and the perceived optimal use declared by academics?

The Wilcoxon paired samples tests (Table 1) were computed to identify the differences between the effective use of technology and what the academics and students consider to be optimal use. The results showed that the optimal use is significantly lower than the actual use for all the contexts (teaching, administrative tasks, and non-job activities), except for research activities, the differences for this context being not significant. Teachers consider closer to optimum the screen time allotted to research activities probably because surveying the specialty literature evidently implies the use of technological devices. The most striking overuse (as considered by the participants) is for administrative tasks – this may be due to the respondents’ perception that a teacher should only teach. However, the respondents may be overusing technology as they declare using it too much for non-academic activities, too. Summing all the hours reported by teachers it almost seems that they spend all their active hours in contact with technology which indicates an over-estimation of the time. Regarding how accurate the estimation is, what is relevant is that they prefer shorter duration of technology use.

**Table 1:** Differences between effective use and optimal use of technology (Wilcoxon test) (Mean of hours/day).

How many hours a weekday do you spend/ consider optimal for using technology for:		*M*	*SD*	*Z*	*sig*
Teaching activities^1^	Effective use	3.85	3.89	6.52	<0.001
	Optimal use	2.50	2.26		
Research activities^1^	Effective use	2.54	3.15	0.68	0.493
	Optimal use	2.27	2.29		
Administrative tasks^1^	Effective use	4.89	3.92	9.87	<0.001
	Optimal use	2.90	2.94		
Non-job activities^1^	Effective use	4.16	5.86	4.71	<0.001
	Optimal use	2.18	1.8		
Academic tasks^2^	Effective	4.45	2.69	7.06	<0.001
	Optimal	3.63	1.72		
Non-academic tasks^2^	Effective	4.37	2.76	11.22	<0.001
	Optimal	3.05	1.97		

^1^Academics (*N* = 233), ^2^Students (*N* = 435).

The results also showed that both academics (Friedman test) and students (Wilcoxon test) use the technology more for non-academic tasks than for academic tasks during weekends (Table 2): for academics because four contexts were compared (Teaching activities, Research activities, Administrative tasks, Non-Job activities) while for students two contexts were compared (Academic tasks, Non-academic tasks). Although the use of technology is perceived as high, devices are not always connected to professional objectives, free time activities being supported by technological devices. This extended use of technology in all areas of life may lead to a misperception of excessive use. When most work-related activities involve technology, personal tasks requiring technology may be seen as overwhelming and this may explain why teacher feel they use too much technology during the weekend.

**Table 2:** Differences between the use of technology for academic and non-academic tasks.

How many hours a weekend day do you spend using technology for non-job activities?	*M*	*SD*	*×^2^*	*sig*
Teaching activities^1^	1.583	2.1129	6.199	0.045
Research activities^1^	1.802	2.3931		
Administrative tasks^1^	1.227	1.6065		
Non-Job activities^1^	4.036	3.3046		
	M	SD	*z*	sig
Academic tasks^2^	3.881	2.834	2.341	<0.001
Non academic tasks^2^	5.307	3.475		

^1^Academics (*N* = 233), ^2^Students (*N* = 435).

### Formal and informal rules, expectations, policies about the use of technology

In order to analyze the overload of academics during the typical weekdays, we compared the number of received demands, responded and initiated demands after work program. The Nonparametric Friedman test for repeated Measures (Table 3) showed that the number of received demands is significantly higher than the solved and initiated demands, while the number of demands that the participants responded is higher than the number of initiated demands. Not only that there are significant differences but what is important and stands out is that the work takes place after work hours. Professional deontology or over-responsibilization/consciousness may make teachers self-overwhelmed whereas students seem to be more able to prioritize their own well-being.

**Table 3:** Differences between time spent receiving, responding, and initiating demands during weekdays.

How many work-related/university-related demands did you (1 received) (2 responded) (3 initiated) after hours in an average week?	*M*	*SD*	*×^2^*	*sig*
Received^1^	6.43	9.45	138.394	<0.001
Responded^1^	4.64	7.66		
Initiated^1^	3.00	6.97		
Received^2^	3.64	4.31	237.709	<0.001
Responded^2^	2.21	3.02		
Initiated^2^	1.48	1.94		

^1^Academics (*N* = 194), ^2^Students (*N* = 359).

Both students and teachers declare that receive more work requests than they initialize which may be caused by a bad management on the teachers’ part and a great number of requests both from their leaders and students. When students are not satisfied with their schedule, they may feel overwhelmed because of specific time constraints or their incapacity of managing their time. The family’s hyper-protection could lead students to a behavior centered on demanding without offering something instead and lack taking responsibility.

To compare the total time spent on the average weeknight versus weekday engaged in work-related demands, the Wilcoxon paired sample test was used, showing a higher amount of time allocated to work during weekend (Table 4).

**Table 4:** Differences between time spent for solving demands.

Please estimate the total time you spent on the average weeknight engaged in….	*M*	*SD*	*t*	*p*
Work-related demands weeknight^1^	1.72	2.04	3.514	0.001
Work-related demands – weekend^1^	2.22	2.67		
Learning-related demands weeknight^2^	2.04	2.82	3.817	0.002
Learning-related demands – weekend^2^	2.68	4.51		

^1^Academics (*N* = 194), ^2^Students (*N* = 359).

### Correlations between technostress and amount in time engaged in using technology and responding to demands in different contexts

Significant Spearman associations were also found between the two aspects of technostress (creators and inhibitors) and the time spent using technology in different context (job and non-job activities, during weekdays or weekends) for the academics (Table 5). Perceived overload was higher for those participants reporting they spend more time involved in teaching activities and personal responsibilities during weekdays. On the other hand, perceived technical support was higher for this category of participants. Overwhelmed teachers declare that they feel they receive technical support only for the teaching activities and that they are not well enough prepared for administrative tasks and technology use is not facilitated. Literacy facilitation and Involvement, as technostress inhibitors were negatively associated with the time spent for administrative tasks, this pattern being nearly the same for weekdays and weekend. An interesting result refers to the positive associations between the time spent engaged in managing work-related demands and techno stressors in general, showing that the higher the time, the higher the levels of stress. As expected, technostress creators correlated negatively with technology self-efficacy and positively with technostress inhibitors. Social support from colleagues and the nearest superior was positively correlated with technostress inhibitors, showing that support could a be stress suppressor. The significant negative association between teaching activities performed during the weekend and IT literacy may show that teachers save the weekend for keeping up to date with the use of technology.

**Table 5:** Associations between technostress and use of technology in different contexts (for academics).

	***Techno stressors***				***Technostress inhibitors***			
	Overload	Invasion	Complexity	Total	Literacy	Support	Involvement	Total
**Use of technology for… (during weekdays)**								
Teaching activities -	0.193**	0.084	0.028	0.146*	0.131	0.187**	0.010	0.142*
Research activities	−0.051	0.166*	0.007	0.026	−0.029	0.061	−0.044	0.021
Administrative tasks	0.078	−0.005	0.131	0.087	−0.268**	−0.028	−0.164*	−0.177*
Personal responsibilities	0.186**	−0.056	0.038	0.087	0.044	0.085	0.033	0.066
**Use of technology for… (during weekend)**								
Teaching activities	0.092	0.172*	0.036	0.116	−0.252**	0.251**	0.040	0.239**
Research activities	−0.036	0.172*	−0.043	0.028	0.048	0.107	0.098	0.036
Administrative tasks	0.044	0.192*	0.065	0.113	−0.148*	−0.058	−0.167*	−0.136
Personal responsibilities	−0.011	−0.017	−0.046	−0.025	−0.061	−0.111	0.050	−0.058
**Time you spent on the average weekday engaged in….**								
Received demands	**0.221** ^**^	0.220**	0.087	0.233**	−0.119	−0.047	0.015	−0.069
Responded demands	0.150*	0.300**	0.142*	0.231**	−0.098	−0.013	−0.052	−0.065
Initiated demands	0.261**	0.316**	0.192**	0.317**	0.014	0.031	0.002	0.031
**Time you spent on the average weeknight engaged in….**								
Work related demands	0.225**	0.358**	0.099	0.285**	−0.021	0.013	−0.059	−0.019
**Time you spent on the average weekend engaged in….**								
Work related demands	0.082	0.324**	0.076	0.183*	0.052	0.077	−0.011	0.046
**Technology self-efficacy**	−0.390***	−0.228**	−0.600***	**−0.459** ^***^	0.227**	0.211**	0.107	0.207**
**Support**								
From colleagues	−0.118	0.054	−0.002	−0.044	0.276**	0.226**	0.155*	0.253**
Nearest superior	−0.005	−0.054	0.031	−0.012	0.170*	0.152*	0.180*	0.197**

*N* = 202, ***p < 0.001, **p < 0.01, *p < 0.05. Bold values highlight the statistical significant results.

Significant Spearman associations were also found between the two aspects of technostress (creators and inhibitors) and the time spent using technology in different context (academic and non-academic activities, during weekdays or weekends) for students (Table 6). Perceived overload was higher for those students reporting they spend more time involved in non-academic responsibilities during weekdays. The higher the time spent on the average weekday and weekend engaged in managing demands, the higher the perceived level of technostress, seen as invasion and complexity. Both for students and teachers, technostress creators correlated negatively with technology self-efficacy and positively with technostress inhibitors. Social support from colleagues and teachers was positively correlated with technostress inhibitors, showing that support could a be stress suppressor, the correlations were higher for social support for students than for academics.

**Table 6:** Associations between technostress and use of technology in different contexts (for students).

	***Techno stressors***				***Technostress inhibitors***			
	Overload	Invasion	Complexity	Total	Literacy	Support	Involvement	Total
**Use of technology for… (during weekdays)**								
Academic activities	−0.052	0.127*	−0.082	0.051	0.022	0.096	−0.034	0.043
Non-academic activities	−0.138**	−0.129*	−0.088	−0.158**	0.070	0.032	−0.020	0.041
**Use of technology for… (during weekend)**								
Academic activities	0.070	0.200**	0.043	0.098	0.022	−0.040	−0.058	−0.013
Non-academic activities	−0.111*	−0.108*	−0.027	−0.111*	−0.013	−0.043	−0.068	−0.038
**Time you spent on the average weekday engaged in….**								
Received demands	142*	0.096	0.097	0.136*	−0.030	−0.058	−0.044	−0.045
Responded demands	0.099	0.116*	0.081	0.123*	0.022	0.012	0.031	0.030
Initiated demands	0.134*	0.139*	0.151*	0.181*	0.063	0.010	0.087	0.064
**Time you spent on the average weeknight engaged in….**								
Univ. related demands	0.078	0.103	0.113*	0.122*	0.041	0.003	−0.002	0.031
**Time you spent on the average weekend engaged in….**								
Univ. related demands	0.125*	0.182**	0.170**	0.201**	0.056	−0.071	0.049	0.025
**Technology self-efficacy**	−0.356***	−0.108*	−0.543***	**−0.397** ^***^	**0.099**	0.158**	**0.015**	**0.102**
**Support**								
From colleagues	−0.057	0.050	0.040	−0.004	0.111*	0.033	0.155**	0.109*
From teachers	−0.149**	−0.037	−0.081	−0.123*	0.346***	0.264**	0.310***	0.363***

*N* = 372, ***p < 0.001, **p < 0.01, *p < 0.05. Bold values highlight the statistical significant results.

### Correlations between technostress and work experience

Work experience and technostress showed very weak correlations, there was only one significant correlation with Techno-complexity (*r* = 0.26, *p* < 0.001). For students, academic standing correlated negatively but the correlations were very weak, with Literacy facilitation (*r* = −0.132, *p* = 0.013) and Involvement facilitation (*r* = −0.124, *p* = 0.019).

### Country differences for technostress

We also aimed to identify possible differences between countries for the main variable of this study, technostress. One-way ANOVA tests were computed to compare data collected from the four countries. Table 7 presents comparisons for two of the technostress creators, techno-overload and techno-invasions, respectively for two inhibitors, literacy facilitation and technical support. Techno-complexity did not show significant differences.

**Table 7:** Differences between countries for technostress and technology self-efficacy.

	Academics						Students					
Country	*M*	*SD*	*F*	*df*	*p*	*Eta^2^*	*M*	*SD*	*F*	*df*	*p*	*Eta^2^*
Technostress creators												
Romania	2.46	0.86	1.586	3,228	0.194	0.024	2.77	0.75	1.659	3,422	0.176	0.013
Germany	2.86	0.69					2.94	0.82				
Finland	2.73	0.53					2.62	0.75				
Norway	2.65	0.84					2.88	0.69				
Techno overload												
Romania	2.38	1.03	7.075	3,228	< 0.001	0.098	2.71	0.90	3.795	3,228	0.011	0.030
Germany	3.18	1.06					3.11	0.88				
Finland	3.22	0.82					2.90	0.98				
Norway	2.82	0.98					3.06	0.69				
Techno invasion												
Romania	2.806	1.065	1.205	3,196	0.309	0.018	3.16	0.98	3.696	3,228	< 0.001	0.029
Germany	2.773	0.833					3.24	0.98				
Finland	2.413	0.894					2.69	0.99				
Norway	2.634	0.955					3.04	0.95				
Technostress inhibitors												
Romania	3.33	0.82	5.377	3,228	< 0.001	0.076	3.11	0.73	0.896	3,228	0.443	0.007
Germany	3.00	0.40					2.98	0.61				
Finland	3.41	0.65					3.22	0.48				
Norway	2.89	0.63					3.08	0.64				
Literacy facilitation												
Romania	3.42	1.00	4.649	3,228	0.004	0.066	3.197	0.859	1.905	3,228	0.128	0.015
Germany	2.78	0.62					2.944	0.802				
Finland	3.51	0.84					3.304	0.586				
Norway	2.98	0.70					3.031	0.804				
Technical support												
Romania	3.75	0.94	15.357	3,228	< 0.001	0.190	3.336	0.831	1.368	3,228	0.252	0.011
Germany	3.86	0.72					3.445	0.800				
Finland	3.82	0.81					3.574	0.608				
Norway	2.79	0.96					3.356	0.857				
Technology self-efficacy												
Romania	4.21	0.54	3.561	3,228	0.015	0.052	4.11	0.67	1.766	3,228	0.153	0.014
Germany	4.09	0.41					3.99	0.60				
Finland	3.98	0.58					3.90	0.64				
Norway	3.90	0.65					4.09	0.64				

For technostress creators there were no overall differences between countries, the Techno-overload dimension was the only significant one, the Games Howell *post hoc* tests showing significant higher levels of perceived overload for Finish academics compared to Romanian academics (mean dif. = 0.84, *p* < 0.001), and for Norwegian academics compared to Romanian academics (mean dif. = 0.44, *p* = 0.041). For technostress inhibitors, the overall score showed significant country differences, the Norwegian academics reporting higher levels of perceived inhibitors (mean dif. = 0.43, *p* = 0.001). The analysis for each type of technostress inhibitors showed significant differences for Literacy facilitation and Technical support. For Literacy facilitation, the results showed that Romanian academics perceive higher levels of facilitation than German academics (mean dif. = 0.64, *p* = 0.038) and Norwegian academics (mean dif. = 0.43, *p* = 0.009). Finish academics reported higher levels of facilitation than German academics (mean dif. = 0.73, *p* = 0.035). For Technical support, we found three significant differences, as follows: Romanian academics report higher levels of support than Norwegian academics (mean dif. = 0.96, *p* < 0.001); German academics report higher levels of support than Norwegian colleagues (mean dif. = 1.06, *p* = 0.003); Finish academics report higher levels of support than Norwegian academics (mean dif. = 1.03, *p* < 0.001).

For technology self-efficacy, we found country differences, Romanian academics having the highest level of efficacy, and Norwegians, the lowest. The Games Howell *post hoc* test showed only one significant difference between Romania and Norway (mean dif. = 0.31, *p* = 0.015).

For the student sample, the results showed no significant differences for technostress creators between countries. However, the Techno-overload dimension was the only significant one, although the *post hoc* tests did not show significant differences, the higher levels of overload were reported by German and Finish students compared to Romanian and Norwegian students. The techno-invasion also showed differences (*post hoc* Hochberg tests), Romanian students reporting higher levels of techno invasion than the Finish students (mean dif. = 0.47, *p* = 0.009). For Techno inhibitors there were no overall differences between countries, no differences were found also for the dimensions of techno inhibitors. There were no country differences for students regarding technostress.

There were no differences in technostress between academic fields, except a small difference for Technical support (*F* = 2.530, *p* = 0.042), Natural and applies sciences academics reporting the highest perceived support. For students, there was no statistically significant difference in terms of technostress regarding their academic field. There were no country differences in technology self-efficacy either, neither for the academic sample not the students one.

### Country differences regarding the number of hours spent using technology

Kruskal Wallis tests were computed to analyze the differences between countries regarding the number of hours spent using technology. The results showed significant differences for all the dimensions, German academics spending more time online for teaching, research and administrative activities during weekdays, while Romanian academics declared they spend more time using technology for teaching and research activities during weekends. On the other hand, Finish academics spend more time using technology for non-job activities (Table 8). Some caution should be taken in trusting the number of hours reported by German participants since the numbers are very high in all categories.

**Table 8:** Differences between countries for the number of hours spent using technology (academics sample).

	Country	*M*	*SD*	*×^2^*	*df*	*sig*
How many hours a weekday do you spend using technology for: job-related (compulsory and non-job compulsory) for: [Teaching activities]	Romania	3.67	3.06	48.047	3	<0.001
	Germany	9.50	4.19			
	Finland	7.68	5.42			
	Norway	1.68	1.85			
How many hours a weekday do you spend using technology for: job-related (compulsory and non-job compulsory) for: [Research activities]	Romania	2.79	2.73	39.402	3	<0.001
	Germany	7.50	4.64			
	Finland	1.87	4.09			
	Norway	1.58	2.09			
How many hours a week day do you spend using technology for: job-related (compulsory and non-job compulsory) for: [Administrative tasks]	Romania	3.68	3.51	43.707	3	<0.001
	Germany	9.42	4.01			
	Finland	8.59	4.56			
	Norway	4.75	2.68			
How many hours a weekend day do you spend using technology for: job-related (compulsory and non-compulsory): [Teaching activities]	Romania	2.23	2.29	37.867	3	<0.001
	Germany	2.00	1.60			
	Finland	1.39	2.59			
	Norway	0.56	0.98			
How many hours a weekend day do you spend using technology for: job-related (compulsory and non-compulsory): [Research activities]	Romania	2.64	2.64	42.276	3	<0.001
	Germany	1.50	1.68			
	Finland	1.03	2.57			
	Norway	0.74	1.22			
How many hours a weekend day do you spend using technology for: job-related (compulsory and non-compulsory): [Administrative tasks]	Romania	1.39	1.64	16.120	3	0.001
	Germany	1.54	1.20			
	Finland	0.83	2.10			
	Norway	1.08	1.34			
How many hours a week day do you spend using technology for non-job activities? (Personal responsibilities/Leisure)	Romania	2.65	2.43	85.351	3	0.004
	Germany	4.63	2.99			
	Finland	13.84	8.43			
	Norway	2.19	4.56			
How many hours a weekend day do you spend using technology for non-job activities? (Personal responsibilities/Leisure)	Romania	3.51	3.15	13.279	3	0.495
	Germany	5.00	4.11			
	Finland	5.50	3.27			
	Norway	4.02	3.27			

Kruskal Wallis tests were computed to analyze the differences between countries regarding the number of hours students spent using technology. There were statistically significant differences for German students using technology more frequently for academic tasks during weekdays, while Romanian students use more frequently technology for academic activities during weekends (Table 9).

**Table 9:** Differences between countries for the number of hours spent using technology (student sample).

	Country	*M*	*SD*	*×^2^*	*df*	*sig*
How many hours a weekday do you spend using technology for: academic activities (compulsory and non-job compulsory)	Romania	4.22	2.63	14.008	3	0.003
	Germany	5.29	2.48			
	Finland	5.02	2.53			
	Norway	4.26	2.98			
How many hours a weekend day do you spend using technology for academic activities (compulsory and non-compulsory)	Romania	4.37	2.95	27.509	3	<0.001
	Germany	3.29	1.84			
	Finland	3.19	2.81			
	Norway	2.70	2.35			
How many hours a weekday do you spend using technology for non-academic activities? (Personal responsibilities / Leisure)	Romania	4.44	2.72	2.295	3	0.514
	Germany	3.87	2.52			
	Finland	4.42	2.72			
	Norway	4.30	3.12			
How many hours a weekend day do you spend using technology for non-academic activities? (Personal responsibilities/Leisure)	Romania	5.45	3.58	4.902	3	0.179
	Germany	4.45	2.81			
	Finland	5.83	4.00			
	Norway	4.68	2.65			

## Discussion and conclusion

One of our study aims was to better understand the technostress in the context of effective versus optimal use of technology among students and academics. As expected, the effective time spent online is higher than what academics and students consider to be optimal, for all the contexts, both professional and non-professional. The study investigated whether a notable difference between the volume of demands received and those resolved or initiated indicates potential overload. The results confirmed this hypothesis, showing a significantly higher number of received demands than solved or initiated. Also, the study explored potential correlations between technostress and various facets of technology usage, showing positive associations between technostress creators and time spent online, negative correlations between technology self-efficacy and technostress creators and positive correlations with technostress inhibitors. Several country differences were explored, showing higher level of technology self-efficacy for Romanian academics, higher usage of technology during weekdays for work and academic activities for German academics, and more frequent use of technology during weekends for both Romanian students and academics. No notable country differences were identified for technostress.

### Differences between students and academics in technostress and techno inhibitors

Technostress Variations: Our findings reveal significant variations in technostress experiences between students and academics. Academics, particularly those with administrative roles, reported higher levels of techno-overload compared to students, primarily due to the multifaceted nature of their job responsibilities (Tarafdar et al., 2015; Hauk et al., 2019). This is evident in their reported overuse of technology for administrative tasks, with a mean of 4.89 h per day versus an optimal use of 2.90 h (Table 1), underscoring the intensity of techno-overload in their professional lives. Academics often face higher levels of technostress, particularly in techno-overload and techno-invasion, being involved in a diverse range of activities including teaching, research, and administrative tasks, each with its own technological demands (Ragu-Nathan et al., 2008; Hauk et al., 2019). The high expectations and multitasking nature of their job can lead to a sense of constant pressure and the feeling of being overwhelmed by technology (Tarafdar et al., 2015). Students generally experience lower levels of technostress compared to academics. Their primary interaction with technology is often related to learning and personal tasks. However, they are not immune to technostress, especially when technology is used intensively for educational purposes or when they face difficulties in using new learning technologies (Upadhyaya and Vrinda, 2021).

Techno-Invasion and Work-Life Balance: A distinct aspect of techno-invasion was observed among academics. The expectation of continuous availability, reflected in the significant difference in the number of work-related demands received after hours, demonstrates the invasion of technology into personal time, contributing to work-life imbalance. Academics often struggle with techno-invasion, as their professional and personal lives become increasingly intertwined due to technology, leading to after-hours work demands. In the light of P–T fit model (Ayyagari et al., 2011) too much use of technology creates negative consequences on people life that counteract not only in form of stress levels perceived but also in ineffective activities, lack of well-being and lower level of work satisfaction. Bearing this in mind and analyzing the significant differences reported by our participants on the number of optimal and effective hours of using technology, intervention measures at individual and organizational levels might be necessary.

In contrast, students showed lower levels of techno-invasion, possibly due to more flexible schedules, they typically have more flexibility and control over their time, which might mitigate this effect. Institutional policies regarding work-life balance and expectations for after-hours communication could also explain differences between academics and students and the impact on technostress levels. Academics in institutions with strict or unclear policies about after-hours work might experience higher techno-invasion and overload (Kim and Chon, 2022). In addition, the availability of resources and training for effectively using technology also plays a crucial role. Institutions that provide comprehensive support and training and offer flexibility regarding working hours can help reduce technostress for both students and academics (Gabbiadini et al., 2023; Saleem and Malik, 2023). The emphasis on work-life balance becomes increasingly significant in the context of the growing use of technology, the boundaries between professional and personal life often blur, making it challenging for employees to maintain a healthy balance (Curcuruto et al., 2023).

In order to lower the technostress level, the colleagues’ and managers’ support has a great influence because sharing their own discoveries and advances in technology lead to group learning and venting emotions among colleagues who understand one’s feelings (and even share them) lead to emotional support making the teachers perceive their job as easier and more bearable. Such emotional co-regulation among colleagues are more easily established based on mutual trust relationships. Students can benefit from teacher support and our study identified similar results as previous study in terms of lower levels of technostress while students perceived their teacher support (Saleem et al., 2024).

The use of technology during the weekend is perceived as intrusive for all activities related to work because it shortens the time with one’s family, with friends and for other relaxing activities. Also, personal tasks are transferred to weekend so that these are also perceived as greater effort. Even meeting with colleagues during the weekend may be seen as mutual support, not overtime.

Introducing well-being strategies in everyday life could alleviate the overwhelming feeling of too much technology. A protective factor against technostress is IT resilience, seen as the ability to assimilate factors that could lead to a negative perception of ICT and to bounce back to the initial state of well-being (Klesel et al., 2018). Elements of the IT resilience are self-efficacy in technology use, a positive attribution and attitude towards technology as well as the ability to adapt to new ways of working based on new technology along with the technology-induces stress, and mainly the ability to keep succeeding in face of adversity created by the technological development and the necessity to use it while maintaining the work-life balance. The capacity to manage the technostress generated by the mandatory technology involved in each domain is translated into IT resilience.

Techno-Complexity and Technology Self-Efficacy: Interestingly, our data suggest a correlation between techno-complexity and technology self-efficacy, particularly among academics (Table 5). Those with lower self-efficacy reported higher techno-complexity, aligning with previous research indicating that confidence in one’s ability to use technology can mitigate perceptions of its complexity (Venkatesh et al., 2003). Self-efficacy in technology is the main factor leading to alleviating technostress (Woolston, 2019; Chou and Chou, 2021). In terms of differences between academics and students regarding techno-complexity, academics might experience higher techno-complexity due to the need to use and understand a wide range of technologies. Techno-insecurity can also be more pronounced among academics due to concerns about technological advancements impacting their job security or changing their professional roles. Students’ concerns might be more focused on mastering the technology required for their studies.

Techno-Overload: Academics frequently report techno-overload due to the necessity to stay up-to-date with numerous digital platforms for research, teaching, and administrative work. In contrast, students might experience techno-overload primarily during periods of intense online learning or when balancing technology use for both educational and personal purposes. As mentioned by Upadhyaya and Vrinda (2021) techno-overload and techno-invasion are the most common techno-stressors among students.

Techno-Inhibitors and Support systems: Both students and academics benefit from techno-inhibitors such as literacy facilitation and technical support. However, the level of perceived support varies, with academics generally reporting higher levels of technical support. This difference could be attributed to the more structured support systems available in academic settings, as opposed to the relatively less formalized support structures for students who might rely more on self-help and peer support.

Time spent using technology: A notable difference was observed in the amount of time spent using technology for job-related activities. German academics reported significantly higher usage for teaching, research, and administrative tasks during weekdays, suggesting a higher integration of technology in their professional lives compared to academics from other countries. For students, German and Finnish students reported higher technology usage during weekdays for academic tasks, indicating a possible higher reliance on digital tools in their educational processes.

In discussing the interplay of technostress, technology self-efficacy, and country differences among academics and students, it is essential to intertwine the findings of our data analysis with insights from the literature review. The concept of technostress, as defined by (Brod, 1984) highlights the struggle to adapt to new computer technologies. Our data revealed nuanced differences across countries, reflecting varying cultural attitudes towards technology and work-life balance.

### Country differences

For academics, the analysis uncovered significant country differences in perceived technostress. Particularly, Finnish and Norwegian academics reported higher techno-overload compared to their Romanian counterparts (mean difference of 0.84 and 0.44, respectively, *p* < 0.05), suggesting a more intense pressure in these countries to adapt to technological advancements in educational settings. This aligns with the literature indicating a high pace of technology development as a source of stress (Ragu-Nathan et al., 2008; Tarafdar et al., 2015) In terms of technostress inhibitors, Norwegian academics perceived lower levels compared to other countries, especially in literacy facilitation and technical support. This contrasts with Romanian academics who reported higher levels of these inhibitors, indicating better adaptation mechanisms in place, possibly due to a more relaxed cultural approach to technology use and work-life balance. Also, one should consider the cultural differences in perception regarding overload. Several previous studies have shown that Romanian employees tend to work overtime and feel natural to work without clear work-life balance (Igret et al., 2016; Tziner et al., 2019).

The technology self-efficacy findings showed Romanian academics to have the highest level of efficacy, with a significant difference from Norway (mean difference = 0.31, *p* = 0.015). This suggests that self-confidence in using technology is higher in environments where technology is more seamlessly integrated into academic life, as also indicated by the higher usage of technology for both work and leisure activities in Romania.

For students, the data revealed no significant differences between countries in terms of technostress creators. However, there were notable differences in techno invasion, with Romanian students experiencing higher levels than their Finnish counterparts (mean difference = 0.47, *p* = 0.009). This could be reflective of the different educational systems and the extent to which technology is embedded in the learning process.

In terms of technology use, German academics and students reported higher usage during weekdays for work and academic activities, respectively, while Romanian students and academics used more technology during weekends. This might reflect cultural differences in work and study habits, with the German context possibly emphasizing a more structured weekday focus and the Romanian context a more fluid integration of work and leisure activities throughout the week.

The findings also showed no significant differences in technostress across academic fields, suggesting that the impact of technology on stress levels is more related to the individual and cultural context rather than the field of study. This is an important insight as it indicates that interventions to mitigate technostress should be tailored to cultural and individual factors rather than being field-specific.

Individual differences in adaptability to new technologies and personal technology skills can influence the experience of technostress, age and experience are important personal factors explaining the perceived technostress, however in our study work experience and technostress showed very weak correlations. Previous studies showed that older academics might experience higher technostress due to possible lower digital literacy compared to their younger counterparts (Hauk et al., 2019). For students, technology usage is often more intuitive, but the stress might arise from the pressure of using these tools to obtain academic performance. Academics and students with higher digital literacy and a positive attitude towards technology are likely to experience lower levels of technostress.

### Limitations and future research directions

Some limitations are under question in this research. Firstly, the cross-sectional design of our study does not allow causal conclusions. Therefore, future research could be conducted with a longitudinal design to study the variation in the use of technology and its relationship with technostress over time. Secondly, due to the cross-cultural study, data collection led to a relatively unbalanced sample concerning academic field, type of university, educational level, therefore the generalizability of the results is limited. In addition, the sample size was different and smaller for some countries. The small number of academics to represent all the HEI’s structures constituted a limitation. Another limitation refers to the measure used for the time spent online. Academics and students estimated the number of hours spent online in different contexts therefore we measured the perceived time spent online instead of an objective measure. Therefore, there are several limitations and challenges to consider, such as: subjectivity of perception (people’s perception of time is highly subjective and can be influenced by numerous factors, such as their level of engagement, interest, and personal biases), memory recall issues (memory could be unreliable, especially for routine or unremarkable activities like everyday internet use), variability in online activities (variability can make it hard to measure and compare time spent across different types of online activities), social desirability bias (participants might underreport or overreport their online time due to social desirability bias), lack of temporal reference points (it could be difficult for participants to accurately estimate the duration of their online activities) or technological literacy (the level of technological literacy among participants can influence their online behavior and, consequently, their perception of time spent online) (Barthel et al., 2020; Marciano and Camerini, 2022). A future study could use more objective measures such as digital tracking of actual time spent online, given the fact that studies relying on self-reported data can lack precision. The inclusion of more diverse samples, and the potential use of mixed methods to triangulate data and validate self-reported information could also be beneficial to the study.

### Implications and contributions

Our study’s findings can contribute to literature on multiple levels. The study enhances the existing understanding of technostress by extending the research to include students and academics, most of previous studies being focused only on samples of employees. Relating optimal use versus effective use of technology to technostress provides a more objective way to study techno overload in academics. Received demands especially after working hours are a reality in HEI domain and are perceived as techno-invasion and techno-overload both by students and teachers. A strong result and new contribution is the fact that use of technology is perceived as more than optimum not only for job tasks but also for nonjob tasks indicated the high degree digitalization penetrated our everyday activities. At organizational level, the study highlights the fact that the psychological burden of intensive technology use, such as technostress, could overshadowed the potential advantages these digital tools offer to everyday teaching and learning practices, if the overload is too high. Therefore, also given the significant correlation with organizational support, is crucial to counteract the stressors associated with heavy use of the technology. The establishment of a supportive organizational environment and effective welfare mechanisms is vital in reducing technostress and role stress among academics and students. The cross-cultural approach is one of the most important contributions of our study, despite the small differences found. While previous studies showed that technostress is prevalent among various groups, impacting people across different organizational roles, professions, and cultures (Wang et al., 2008), our study found limited differences, showing that after the pandemic individuals from different cultures experienced similar patterns of technostress.

Nonetheless, despite the needed caution in interpreting the results, these findings could be used to develop further research and support interventions within European countries. Technostress levels differ based on an individual’s experience with technology usage. Consequently, higher education management should implement ICT trainings for academics and students to increase not only their levels of technology self-efficacy and well-being, but also their productivity (Chen, 2015; Tarafdar et al., 2015). As a result, it is crucial for management institutions to identify academics and students who are experiencing technostress and offer appropriate support to help them manage it. Managers must understand how Information and Communication Technologies (ICTs) impact academics and students, recognizing that technostress varies among individuals and work contexts. This awareness is crucial for implementing appropriate measures to mitigate technostress (Fernández-Fernández et al., 2023).

Regarding theoretical contributions, this research adds value to the existing literature on technostress by providing a more profound understanding of its role and impacts. The study has addressed the gap in the literature about technostress and the effective use of technology in academics (Tarafdar et al., 2015; Upadhyaya and Vrinda, 2021) and also in students. While previous research on the topic focused mainly on employees (Truța et al., 2023), the present study shows that students can also experience technostress as they frequently interact with technology in the academic environment.

Is also revealed that for students technostress creators (overload and invasion) are negative associated with non-academic use of technology while using technology for academic task is perceived as techno invasion, these findings shading more light on the subject then it was acknowledged before (Qi, 2019).

HEI’s representatives should recognize various technostress factors, including work overload, intrusion into personal life, uncertainty from continuous technology updates, complexity, and perceived insecurity in using ICTs, as these factors could directly influence academics performance and, consequently, the organization’s success.

## Conclusion

The findings suggest that the perceived optimal use of technology is significantly lower than the actual use for all the contexts, with overuse of technology being associated with technostress both in students and academics. Such findings suggest that technostress creators may include aspects regarding the effective duration of time using technology at work or for work purposes and not only the subjective perception of the difficulty of coping with new technologies at work. Our results also showed that technology self-efficacy and social support from colleagues and teachers could act as stress suppressors in individuals’ relationships with technology at work. In societies with high levels of digitalization and digital literacy, the support offered by others still plays an important role in coping with technostress both for teachers and for students.

Another important direction in our study regarded country differences in the use of technology and technostress. Even thou we identified significant differences in techno-invasion and technostress inhibitors, in the teacher sample, respectively techno-invasion in the students sample, the findings are not consistent. The topic of cultural differences in adaptation to new technology and in coping with the pervasiveness of technology in HEI should be further discussed.

This study is one of the first studies to approach the dynamic of technology use in HEI by teachers and students from the perspective of technostress. Based on the obtained results, proactive measures to reduce technostress could be implemented, such as offering training to address issues related to technology complexity and uncertainty; monitoring online time to prevent negative interference between work/school and personal time; or promoting a better professional life vs. personal life balance with further positive effects on academic satisfaction and work/learning productivity.

## Data availability statement

The raw data supporting the conclusions of this article will be made available by the authors, without undue reservation.

## Ethics statement

The studies involving humans were approved by Council of the Faculty of Psychology and Education Sciences, Transilvania University of Brasov. The studies were conducted in accordance with the local legislation and institutional requirements. The participants provided their written informed consent to participate in this study.

## Author contributions

A-MC: Conceptualization, Resources, Software, Supervision, Validation, Writing – original draft, Writing – review & editing. LD: Investigation, Project administration, Supervision, Validation, Writing – original draft, Writing – review & editing. CT: Conceptualization, Investigation, Methodology, Project administration, Supervision, Writing – original draft, Writing – review & editing. CM: Formal analysis, Investigation, Software, Validation, Visualization, Writing – review & editing. RH: Data curation, Investigation, Resources, Validation, Writing – original draft. LN: Conceptualization, Project administration, Writing – original draft. NN: Formal analysis, Investigation, Project administration, Writing – original draft. OV: Conceptualization, Data curation, Methodology, Project administration, Writing – original draft. AR: Formal analysis, Investigation, Methodology, Supervision, Writing – review & editing. TT: Formal analysis, Investigation, Visualization, Writing – original draft. EG: Formal analysis, Investigation, Methodology, Writing – original draft. MD: Formal analysis, Investigation, Methodology, Writing – original draft. DU: Investigation, Methodology, Project administration, Writing – original draft, Writing – review & editing. SW: Formal analysis, Investigation, Methodology, Writing – original draft. MP-I: Resources, Supervision, Writing – review & editing.
